# Carbon-Based Composites with Biodegradable Matrix for Flexible Paper Electronics

**DOI:** 10.3390/polym16050686

**Published:** 2024-03-02

**Authors:** Jerzy Szałapak, Bartosz Zdanikowski, Aleksandra Kądziela, Sandra Lepak-Kuc, Łucja Dybowska-Sarapuk, Daniel Janczak, Tomasz Raczyński, Małgorzata Jakubowska

**Affiliations:** 1 Institute of Mechanics and Printing, Faculty of Mechanical and Industrial Engineering, Warsaw University of Technology, 00-661 Warsaw, Poland; aleksandra.kadziela.dokt@pw.edu.pl (A.K.); sandra.kuc@pw.edu.pl (S.L.-K.); lucja.sarapuk@pw.edu.pl (Ł.D.-S.); daniel.janczak@pw.edu.pl (D.J.); tomasz.raczynski.dokt@pw.edu.pl (T.R.); malgorzata.jakubowska@pw.edu.pl (M.J.); 2 Central Laboratory, Centre for Advanced Materials and Technologies (CEZAMAT), 02-822 Warsaw, Poland; bartosz.zdanikowski.stud@pw.edu.pl

**Keywords:** biodegradability, sustainable electronics, printed electronics, paper electronics, ethyl cellulose, screen printing, flexible electronics, carbon-based composites

## Abstract

The authors explore the development of paper-based electronics using carbon-based composites with a biodegradable matrix based on ethyl cellulose and dibasic ester solvent. The main focus is on screen-printing techniques for creating flexible, eco-friendly electronic devices. This research evaluates the printability with the rheological measurements, electrical properties, flexibility, and adhesion of these composites, considering various compositions, including graphene, graphite, and carbon black. The study finds that certain compositions offer sheet resistance below 1 kΩ/sq and good adhesion to paper substrates with just one layer of screen printing, demonstrating the potential for commercial applications, such as single-use electronics, flexible heaters, etc. The study also shows the impact of cyclic bending on the electrical parameters of the prepared layers. This research emphasizes the importance of the biodegradability of the matrix, contributing to the field of sustainable electronics. Overall, this study provides insights into developing environmentally friendly, flexible electronic components, highlighting the role of biodegradable materials in this evolving industry.

## 1. Introduction

In 2023, the printed electronics industry experienced significant growth and development. This technology is no longer just a concept for the future but is already being used in many commercial applications. The development of electronics has led to the need to create new types of devices that are flexible, disposable, and conform to shape. To achieve this, a range of techniques known from the printing industry has been adapted. For example, with the decline in the market for printed newspapers and books, more and more printing companies around the world are adjusting their operations and adopting new, conductive materials. Currently, electronic printing techniques are commonly used in OLED lighting, healthcare, automobiles, and Industry 4.0, offering new possibilities in remote patient monitoring, modernization of car interiors, and intelligent packaging. According to ResearchAndMarkets.com [1], the value of the printed electronics industry in 2020 was $7.8 billion and was expected to rise to $20.7 billion in 2025. However, in their August 2022 report, they confirmed that “the global size of the printed electronics market reached $53.5 billion in 2021”. In the new report, the publisher expects the market to reach a value of $130.8 billion by 2027 [1,2]. Such a rapid increase is also confirmed by IDTechEx [3] and Transparency Market Research Inc. [4]. This growth is driven by increasing demand for wearable devices and display screens, as well as innovations in packaging and labels, flexible screens, and interactive books and posters.

Due to the growing market for printed electronics and electronics in general, the management of electronic waste is becoming increasingly important. Traditional waste processing methods, such as landfilling or incineration, are particularly non-ecological in the case of electronics, and may lead to the contamination of soil, water, or air [5]. When developing devices whose processing and recovery will have a minimal impact on the environment, every stage of their production must be considered. These factors include the materials, which should be biodegradable, compostable, or recyclable, the methods used for their processing, which should not significantly worsen these properties, and the processes, which should not produce toxic waste and leave a large environmental footprint. Moreover, devices manufactured in this way must be competitively qualitative and economically comparable to those already on the market. Therefore, the development of so-called sustainable electronics is becoming increasingly important [6]. It often takes into account biodegradability and compostability standards in accordance with commonly used standards, such as EN 13432 requiring 90% decomposition after 12 weeks or 90% biodegradation after 6 months in packaging, the more general EN 14995 concerning polymers in general, or ISO 17088 related to the compostability of polymers [7,8,9].

These standards are particularly important in the development of single-use or limited-use electronics. So far, the main focus has been on their flexibility and stretchability [10,11,12]. Currently, increasing attention is being paid to systems that can be prepared on easily disposable paper substrates [13,14], or substrates made of biodegradable or compostable polymers [15,16]. Among these polymers, materials based on bio-derived monomers are of particular interest, with a focus on polylactic acid (PLA) and polysaccharides (cellulose and its derivatives, chitosan). Many studies are being conducted to improve their parameters, especially focusing on mechanical properties and biodegradability [17,18,19,20], or their use in sustainable electronics, both as substrates and as components of conductive layers [16,21,22,23,24]. Particularly important are studies related to disposal, partly due to emerging reports on limitations associated with the disposal of PLA-based waste. For example, Royer et al. point out the limitations in the decomposition of PLA in the marine environment and emphasize the need for appropriate management of the removal of compostable plastics, highlighting the discrepancy between compostability and biodegradation in the environment, suggesting that calling compostable plastics “biodegradable” might be misleading [25]. Some of these polymers require specific conditions of industrial composting, which potentially increases the cost of their use. This gives rise to the idea of recycling biodegradable and compostable polymers for their repeated use before disposal [26].

The rapid development also pertains to the conductive composites applied to these substrates. Mainly two approaches are being considered. The first is the recycling of metals used as the functional phase after the degradation of the substrate and the composite matrix [21]. The second is the composting or biodegradation of the electronic system, after the potential removal of attached chips. It is then important to consider whether the degradation is partial or complete and whether the degradation residues are harmful to the environment. Doubts arise, for example, in studies of biodegradable composites containing a metal functional phase, such as in [27,28]. Such composites should ideally be subjected to recycling, as described in [21]. Conductive materials that will not be recycled and have a minimal negative impact on the environment are composites based on carbon particles (e.g., graphene) [29,30,31]. The main advantage of these types of electronic systems is the reduction of responsibility on the consumer’s part. Although carbon-based materials allow for much lower conductivity than metal particles, they are already used for the preparation of heaters, pressure sensors, etc. 

Apart from disposal issues, for materials of this type, parameters related to printability (e.g., rheology), adhesion to printed substrates, and electrical conductivity are important. In the case of carbon-based composites with biodegradable polymers, in [30], a single screen-printed layer did not achieve electrical conductivity. It was necessary to have at least four printed layers in the case of the patterns dried at room temperature, and six layers when dried at 120 °C, to obtain a sheet resistance (Rs) of 5.1 × 10^3^ and 1.8 × 10^4^ Ω/sq, respectively. In [31], the use of poly(butylene adipate-co-butylene terephthalate) (PBAT) as a matrix, with 40 wt% graphene content as the functional phase, achieved a conductivity of the material at the level of 338 S/m (equivalent to a resistivity of about 0.003 Ωm). However, it should be noted that there is no mention in the publication of printing such polymers, and based on the authors’ experiences, it is difficult to talk about carbon composites allowing for printing with such a high graphene content [32]. On the other hand, in [29], the Rs of the layer was 100 Ω/sq, although there is no information about the ink or paste used, but it is known that the layer thickness was 100 µm, which means a resistivity level of 0.01 Ωm. 

In the present article, prepared carbon composites with a biodegradable matrix made using ethyl cellulose (EC) polymer are presented that allow for achieving a comparable resistivity to other carbon-based biodegradable materials with just one screen-printed layer. The material may be cured in temperatures as low as 60 °C. The best compositions are characterized by good adhesion to paper substrates and resistance to cyclic bending, which provides an interesting solution for applications in flexible single- and multiple-use printed electronics.

## 2. Materials and Methods

### 2.1. Materials

**Figure 1:**
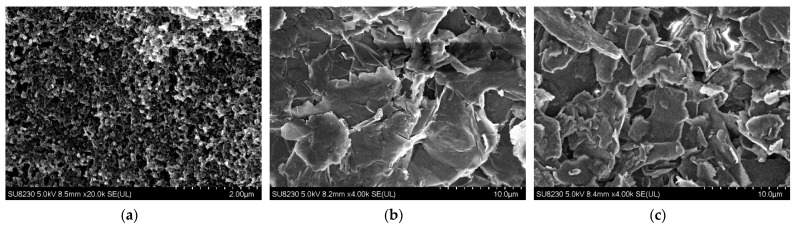
SEM pictures of composites with (**a**) carbon black, (**b**) graphene nanoplatelets, and (**c**) graphite.

For the functional phase of the produced composites, three types of materials were chosen. The first is graphite purchased from Termoplastik, Bydgoszcz, Poland, in the MG 1596 variant. Graphite (Gr) has been extensively studied in terms of electrical parameters [33,34,35]. The second tested material was conductive carbon black (CB) purchased from Black Diamond Material Science Co., Ltd., Hefei, Anhui, China as BeaBlack^®^SCT. Carbon black, as a conductive material, has also been described in many publications, including [36,37,38,39]. The last material was type C graphene nanoplatelets (GNP), commercially purchased from CheapTubes, Grafton, MA, USA. They were selected based on previous research conducted by our research team [40,41]. Materials printed on the substrates are shown in Figure 1.

As a biodegradable matrix for the tests, ethyl cellulose 48.0–49.5% (*w*/*w*) ethoxyl basis purchased from Sigma Aldrich, Burlington, MA, USA was chosen. Ethyl cellulose (EC) is a hydrophobic derivative of cellulose with rigid main chains, in contrast to hydrophilic starch or cellulose [42]. As a solvent, dimethyl succinate was used, which is an ester of dicarboxylic acids that are easily biodegradable and non-toxic. This material is well-known in the printing industry. Ethyl cellulose is a derivative of cellulose, which is naturally biodegradable. Similarly, dimethyl glutarate is a biodegradable ester. On this basis, it can be assumed that the matrix will be a biodegradable material.

For the test substrates, two types of paper were used: 80 g/m^3^ elementary chlorine free (ECF) bleached wood-free pulp paper produced by POLjet, Kwidzyn, Poland, and a similar, 100 g/m^3^ color copy paper produced by Mondi, Świecie, Poland. For reference, test prints were also made on Polyethylenterephthalat (PET) film gloss PC-811 0.175 mm produced by Longhua, Longhua City, China. 

### 2.2. Sample Preparation

In the present study, the impact of four factors on the properties of printed layers was examined: the weight concentration of ethyl cellulose in the solvent, the type of functional phase used, the weight concentration of this phase, and the curing temperature of the prepared composite. These samples were printed on three different substrates. The parameters are presented in Table 1. To limit the number of samples, it was decided to use an appropriate fractional factorial method, in this case, the Taguchi method [43]. It allows for reducing the number of produced samples while enabling the selection of such parameter values to obtain the best samples for each of the studied properties. It is worth emphasizing that in addition to the 18 composites selected based on the fractional factorial method, three additional composites based on the obtained results were prepared: 8% wt. CB in an 8% ethyl cellulose solution, 6 wt%. CB in an wt8% ethyl cellulose solution, and 10 wt%. GNP in a 10% ethyl cellulose solution.

The production of the samples began with the creation of carriers for the functional phase. EC was dissolved in dimethyl succinate. Measured amounts of EC were placed in a vessel and initially mixed with a spatula. Then, to ensure the homogeneity of the material, the samples were mixed on a magnetic stirrer at 150 rpm and left for 48 h. The weighed functional phase was added to the prepared polymer, and after initial mixing with a spatula, the composite was homogenized using a Kakuhunter Planetary Speedmixer by Shashin Kagaku Co., Ltd., Kyoto, Japan. Due to the high density of the pastes containing carbon black (CB), the composite was additionally rolled on a three-roller mill 80E/0413 by EXAKT, Norderstedt, Germany with a gap setting of 10 µm. 

The dynamic viscosity of composites of the prepared composites was examined using a Brookfield RS CPS+ cone-plate rheometer, Middleboro MA, USA, cone spindle C50-1. The measurements were conducted at 25 °C, set using the Polyscience ultrathermostat, Niles, IL, USA. The viscosity value was measured for a shear rate ranging from10 to 500 s^−1^.

The next step was screen printing the pastes onto selected substrates using the C920 device from AUREL Automation Division, Modigliana, Italy and a 68T polyester mesh screen. Each of the samples was cured for 20 min in an IN110 chamber dryer by Memmert, Schwabach, Germany.

## 3. Results and Discussion

### 3.1. Viscosity Measurements

In printed electronics, a composite (implicitly a polymer), by definition, means a material created by the close combination of at least two chemically different materials (phases: functional and matrix) in such a way that, despite a clear separation boundary between them, there is a good and continuous connection of the components and an as even as possible distribution of the functional phase in the matrix. The heterophase materials used in this article consist of a functional phase in the form of carbon particles (CB, GNP, graphite) and a matrix in the form of an ethyl cellulose polymer. The content, type of functional phase, as well as the homogenization method significantly affect the material’s viscosity and rheological behavior. 

**Figure 2:**
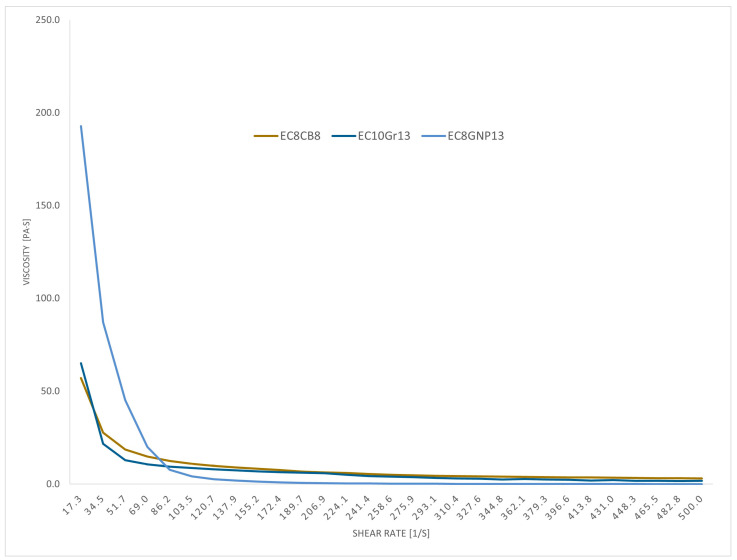
Dependence of viscosity on the shear rate in the representative composite materials.

Screen-printable materials should be characterized by shear-thinning behavior. It is necessary to obtain materials whose viscosity decreases as the shear rate increases, which makes the materials printable and enables obtaining good quality patterns. The viscosity was measured for all tested composites. The viscosity for a shear rate of 18 [1/s] and for a shear rate 100 [1/s] is shown in Table 2. The viscosity of composites containing 13% carbon black is shown in the table with the value 0. Their measurements were impossible to conduct due to exceeding the measurement range of the device. The materials were too viscous, almost powdery. The dependence of viscosity on the shear rate in the representative composite materials is shown in Figure 2. Additional figures may be found in the Supplementary Materials. 

To ensure proper spreading on the screen during printing, according to the authors of [44], pastes for use in thick-film technologies should have a viscosity in the range of 10–70 Pa·s at a shear rate of about 10–20 [1/s]. In the case of the developed composites, most exceed these values, but all except those containing 13% CB were successfully applied to the substrates and further measurements were performed on them. This is due to the fact that the developed materials are characterized by shear thinning, as shown for the exemplary compositions in Figure 2. The viscosities of all pastes were reduced for a shear rate of 100 [1/s]. The highest obtained viscosity value of 40 Pa·s at a 100 [1/s] shear rate was achieved for EC10 GNP10, for which it was a decrease from the initial 166.9 Pa·s. All tested composites, after printing on the substrate, created patterns of good quality. The lack of uncontrolled material flow on the substrate after the stresses (directly related to the printing process) are released allows us to determine that the tested materials do not have thixotropic properties.

Among the prepared composites, those with the highest viscosity posed the greatest challenges in the printing process. Materials with a viscosity that was immeasurable on the available rheometer did not allow for application to substrates. As the concentration of the polymer in the solution increased, as well as with the increasing content of the functional phase, the viscosity of the pastes increased. The type of functional phase had the greatest impact on the rheological parameters—graphite, characterized by the highest density, allowed for obtaining the lowest viscosity values. On the other hand, pastes containing CB were characterized by very high viscosity, resulting from their low density and greater specific surface area. 

### 3.2. Electrical Measurement and Bending

**Figure 3:**
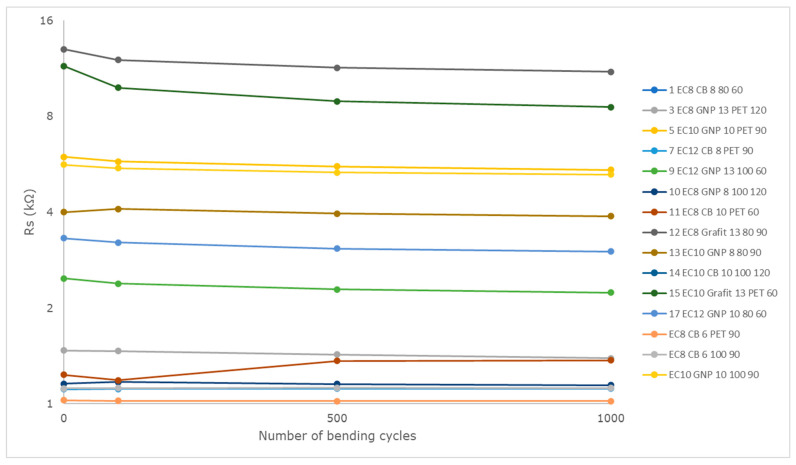
Impact of bending on sheet resistance.

After printing all the measurement paths (for each variation, the series consisted of at least *n* = 30 samples), electrical measurements were conducted using a GW INSTEK GDM-8341 multimeter, Montclair, CA, USA. The prepared samples were then subjected to 1000 bending cycles with a varying bending radius from ∞ to 5 mm. The bending was performed on a single-column Cometech QC 548 tensile testing machine, Taichung City, Taiwan, at a testing speed of 800 mm/min, and the resistance measurement of the paths was conducted after 100, 500, and 1000 cycles, respectively, as shown in Figure 3. Table 3 presents the selected measurement variants, indicating the type of composite, curing temperature, and type of substrate. The results of the resistance measurements and their changes after 1000 cycles of bending are also presented.

The type and then the concentration of the material have the most significant impact on the resistance parameters. Graphite, at such low concentrations, performed the worst in terms of resistance. In the case of graphene, a decrease in resistance can be observed with an increase in the concentration of the functional phase in the layer. Such a clear relationship is not present in the case of CB, but here, the rheological parameters of the composite and the difficulties in applying it to substrates using screen printing should be taken into account.

Another issue is the rather significant spread in the resistance results. For most samples, it is within 20% of the initial resistance, and in no case does it exceed 50%. This means that most composites require improvement in repeatability, but the obtained results allow for determining the expected level of the electrical conductivity of the layers prepared using them. It should also be emphasized that the EC8CB6 composite is characterized by a deviation at the level of 10% of the initial resistance.

Based on the obtained results, it can be concluded that regarding resistance before and after bending, the curing temperature does not seem to have a clear impact on the final resistance of the material, as resistance values vary for different materials cured at the same temperature. Therefore, a curing temperature of 60 °C is sufficient for the used materials in terms of achieving optimal electrical conductivity. The material of the functional phase seems to have a greater impact. Composites with GNP show a smaller change in resistance after bending than those with graphite or CB, suggesting their greater flexibility and better mechanical behavior. The resistance change for all tested composites is shown in Figure 3.

### 3.3. Adhesion

Adhesion tests were carried out using the cross-cut knife method according to ISO 2409 [44,45], with a circular knife produced by Testan and 3M Scotch Tape. A grid of incisions was made on the widest conductive paths, after which, in accordance with the standard’s requirements, the coating was cleaned with a soft brush, and then scotch tape was attached. The tape was removed after 5 min of application. The quality of adhesion is rated on a scale from 0 (best) to 5 (worst rating).

Among the studied layers, samples with a rating of 0 or 1 show better conductive properties and stability after bending than those with higher ratings, although there are cases where materials with a good adhesion quality show a large change in resistance after bending (e.g., EC10GR13).

Higher curing temperatures seem to be beneficial for adhesion quality. Lower curing temperatures may not be sufficient to achieve optimal adhesion—this is particularly evident with CB-containing materials printed on paper: EC8CB8 cured at 60 °C with poor adhesion, rated 4, and EC10CB10 cured at 120 °C, characterized by good adhesion, rated 1.

**Figure 4:**
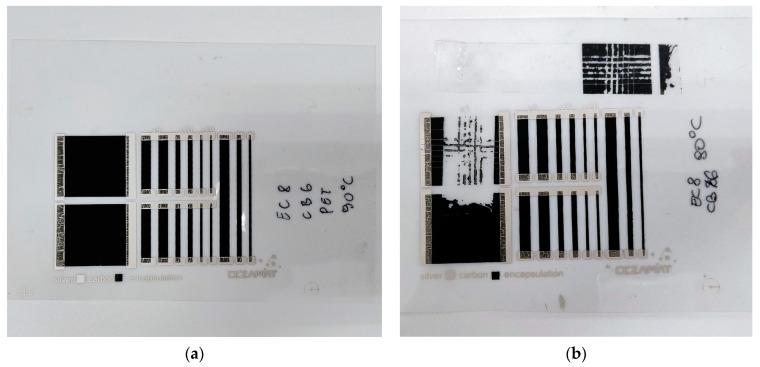
EC8CB6 on PET sample (**a**) before and (**b**) after adhesion test.

Composites on PET substrates generally showed poorer adhesion quality than those on paper. This may be due to the poorer compatibility of the carrier with polymeric substrates, although it is most likely a matter of the higher roughness and porosity of paper substrates. Such a sample on PET is shown in Figure 4a,b.

## 4. Discussion

The concentration of ethyl cellulose in dibutyl glutarate does not seem to have an impact on the mechanical and electrical parameters of the produced composites. No clear influence of the curing temperature on the resistance of the printed layers was also observed. However, temperature does have a clear impact on adhesion—significantly better results were obtained for layers cured at 90 and 120 °C.

Materials with GNP generally show low resistance, which decreases with an increasing concentration of the functional phase. The small change in resistance after bending suggests that they may be suitable for applications requiring flexibility and conductivity, such as flexible electronics. The adhesion quality ratings for GNP composites range from 0 to 1, indicating good adhesion to the substrate. The large resistance spread of some of the GNP-based composites should be reduced. Consideration should be given to making the composite more uniform and breaking up any agglomerates, such as by rolling.

Materials with added graphite require a higher functional phase content to achieve better conductive properties. In future research, a higher graphite content should improve the low conductivity of the layers without much effect on their mechanical performance.

CB-based materials exhibit low resistance, but their behavior after bending varies, which may require further research to understand and optimize their properties for practical applications. Most samples also exhibited low adhesion—for instance, the EC8CB8 sample achieved the lowest resistance of all tested materials, which was 0.80 kΩ/sq while having poor adhesion. However, it is worth noting that the additional EC8CB6 sample made on paper had good adhesion, at level 1, with an Rs below 1 kΩ/sq. Additionally, the standard deviation at the level of 10% of the average surface resistance of the layer is notable. On PET film, the same sample achieved an even lower resistance, but its adhesion received the lowest possible rating. Future research should focus on improving the adhesion and printability of CB-based composites. The results show significant potential for improving the performance of layers made with this material.

The best layer quality was obtained for an 8% ethyl cellulose solution with an 8 wt%. addition of graphene, cured on paper at 120 °C. This is consistent with previous conclusions. It is also worth noting that for this material, a low surface resistance value (Rs = 1.07 kΩ/sq) was also obtained, which was minimally affected by bending.

## 5. Conclusions

In this article, novel, bendable, conductive carbon-based composites were made with a biodegradable ethyl cellulose carrier in a buthyl glutarate solution. These composites are suitable for printed paper electronics and can achieve sheet resistance below 1 kΩ with just one screen-printed layer of approximately 5 µm. The electrical parameters are about two times better in comparison to other printable carbon composites based on biodegradable or compostable polymers for printed electronics applications while being cured in temperatures as low as 60 °C. In some compositions, these materials have decent adhesion to paper and are flexible—not impacted by cyclic bending. At this point of the research, the best compositions have only a 10% standard deviation, which means they may be interesting for commercial use due to decent initial repeatability. Such screen-printable pastes, due to easily scalable technology, may be an important step towards biodegradable printed electronics, hence will allow for growth in single-use electronics without a negative impact on the environment. 

## Figures and Tables

**Table 1:** Selected levels of parameters for the tested samples.

	Level 1	Level 2	Level 3
**EC Weight Concentration [%]**	8	10	12
**Conductive Phase material**	Carbon Black (CB)	Graphite (Gr)	Graphene (GNP)
**Weight Concentration of Conductive Phase [%]**	8	10	13
**Substrate**	Paper 80 g/m^3^	Papier 100 g/m^3^	PET
**Curing temperature [°C]**	60	90	120

**Table 2:** Viscosity of the prepared pastes.

Material	Viscosity (in Pa·s) for Shear Speed of 18 1/s	Viscosity (in Pa·s) for Shear Speed of 100 1/s
EC8CB8	57.1	10.9
EC10CB8	208.5	28.9
EC12CB8	218.3	35.0
EC10CB10	417.1	21.7
EC8CB10	177.2	19.6
EC12CB13	x	x
EC10CB13	x	x
EC8Gr10	4.4	3.6
EC8Gr13	6.2	4.4
EC10Gr8	12.7	7.4
Ec10Gr13	65.1	8.6
EC12Gr8	26.9	12.8
EC12Gr10	29.1	12.8
EC8GNP8	130.4	31.9
EC8GNP13	192.8	4.2
EC10GNP8	96.5	27.7
EC12GNP10	120.5	14.0
EC12GNP13	316.1	39.4
EC10GNP10	166.9	40.0
EC8CB6	86.1	13.8
EC8_CB13	x	x

**Table 3:** Compilation of prepared sample measurement results.

Name	Polymer (Wt%)	Conductive Material	Wt%	Substrate	Curing Temperature [°C]	Resistance [KΩ/sq]	Resistance Drop after Bending	Std. Dev.	Layer Quality
EC8CB8	EC8	CB	8	Paper 80	60	0.80	0%	0.16	4
EC8GR10	EC8	Grafit	10	Paper 100	90	92.00	16%	6.22	0
EC8GNP13	EC8	GNP	13	PET	120	1.25	6%	0.61	1
EC10GR8	EC10	Grafit	8	Paper 100	60	999.00		x	1
EC10GNP10	EC10	GNP	10	PET	90	5.63	9%	0.39	1
EC10CB13	EC10	CB	13	Paper 80	120	x		x	x
EC12CB8	EC12	CB	8	PET	90	1.11	0%	0.10	5
EC12GR10	EC12	Grafit	10	Paper 80	120	999.00		x	1
EC12GNP13	EC12	GNP	13	Paper 100	60	2.45	10%	0.23	1
EC8GNP8	EC8	GNP	8	Paper 100	120	1.07	1%	0.22	0
EC8CB10	EC8	CB	10	PET	60	1.15	−11%	0.20	5
EC8GR13	EC8	Grafit	13	Paper 80	90	10.99	15%	0.74	1
EC10GNP8	EC10	GNP	8	Paper 80	90	4.11	3%	2.03	1
EC10CB10	EC10	CB	10	Paper 100	120	0.94	−2%	0.44	1
EC10GR13	EC10	Grafit	13	PET	60	10.18	26%	2.12	5
EC12GR8	EC12	Grafit	8	PET	120	999.00		x	5
EC12GNP10	EC12	GNP	10	Paper 80	60	2.88	9%	0.60	1
EC12CB13	EC12	CB	13	Paper 100	90	x		x	x
EC8CB13	EC8	CB	13	PET	90	x		x	x
EC8CB6	EC8	CB	6	PET	90	0.90	1%	0.16	5
EC8CB6	EC8	CB	6	Paper 100	90	0.99	0%	0.10	1
EC10GNP10	EC10	GNP	10	Paper 100	90	6.58	7%	0.63	0

## Data Availability

Data are contained within the article.

## Supplementary Materials

The following supporting information can be downloaded at: https://www.mdpi.com/article/10.3390/polym16050686/s1.
