# Supplementary figures and images for: Carbon-Based Composites with Biodegradable Matrix for Flexible Paper Electronics
Source: Polymers (Basel). 2024 Mar 2;16(5):686. doi: 10.3390/polym16050686 (PMC10935179; doi:10.3390/polym16050686)

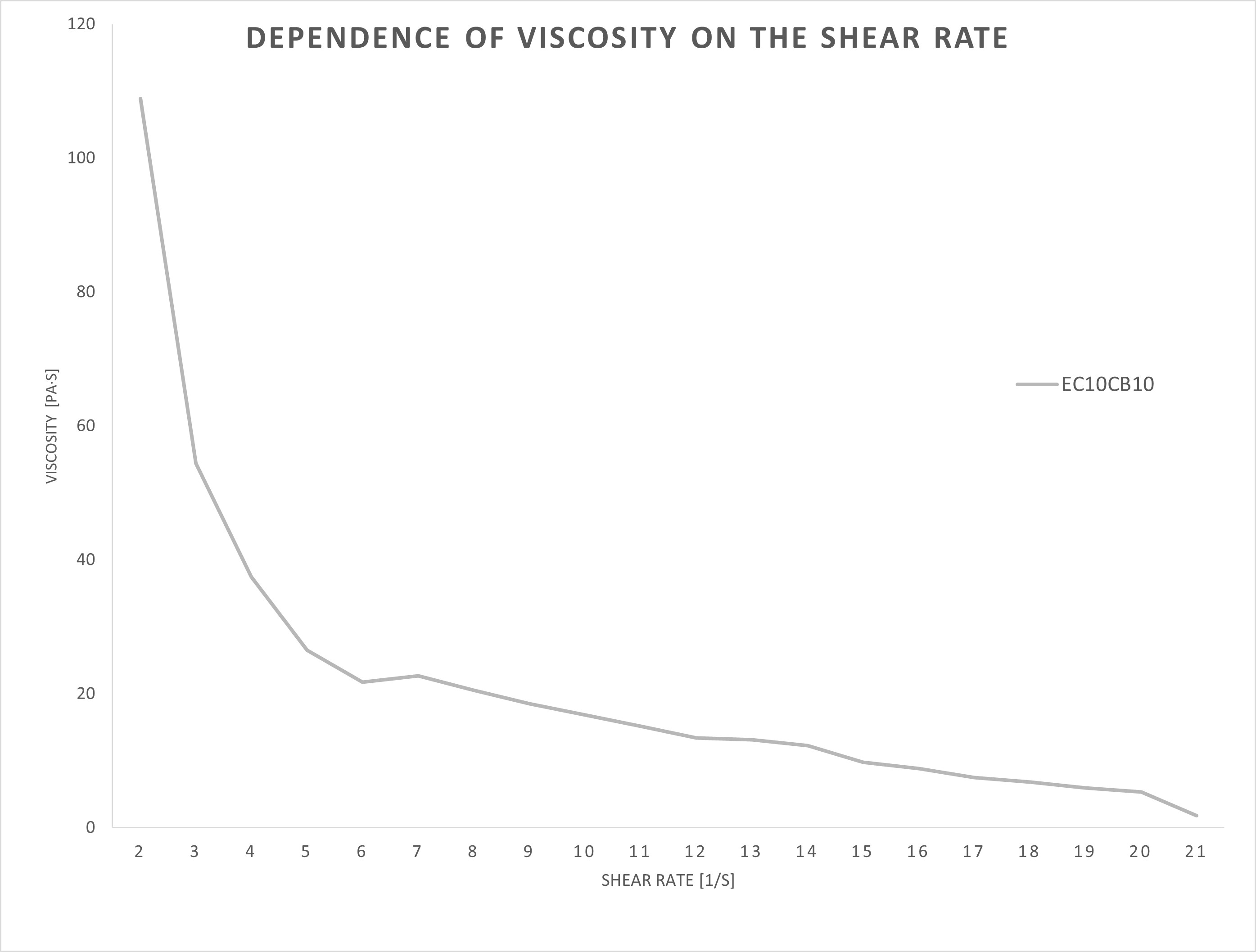

Supplement: Supplementary file 1 [file polymers-16-00686-s001.zip › rheology appendix/EC10CB10.jpg]

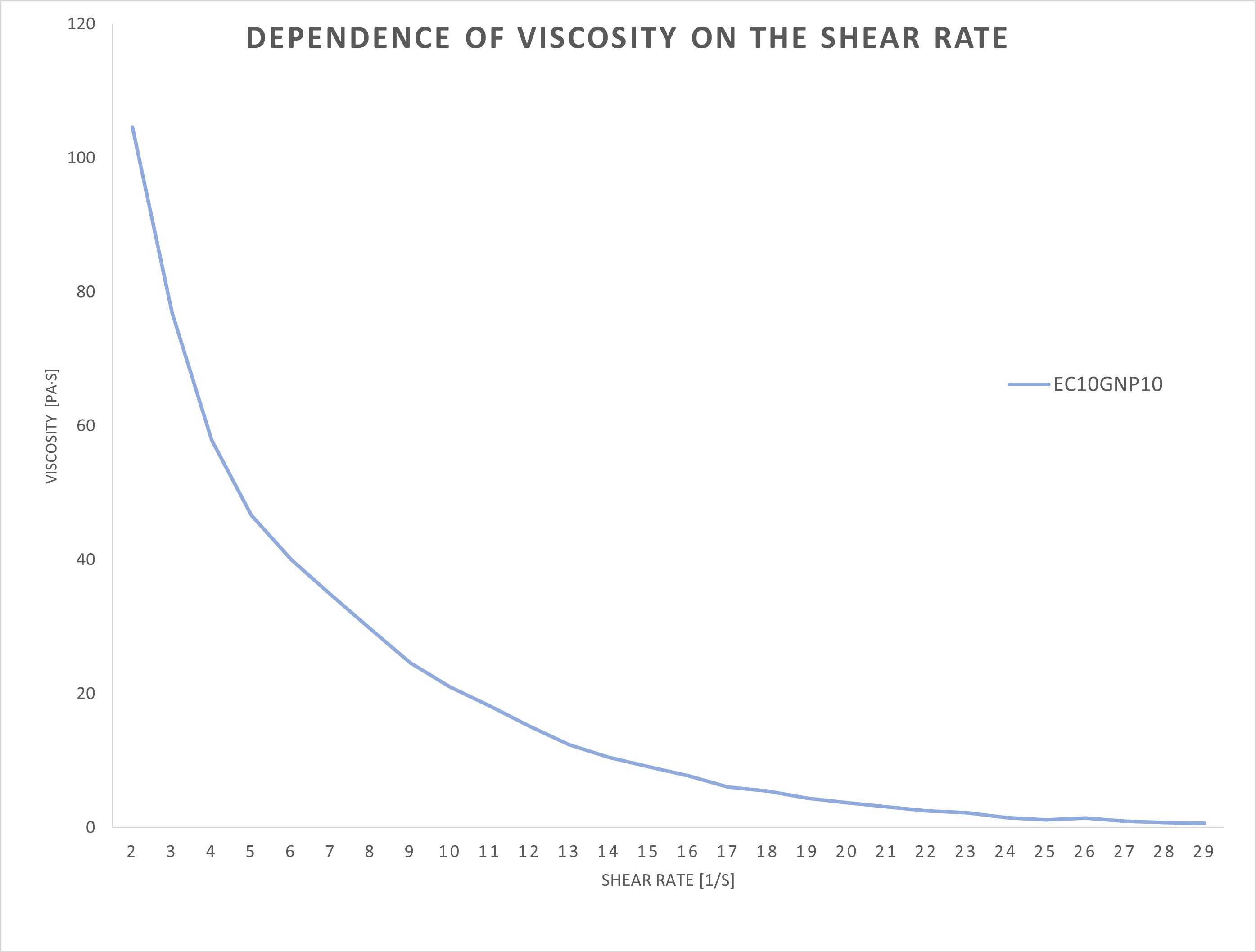

Supplement: Supplementary file 1 [file polymers-16-00686-s001.zip › rheology appendix/EC10GNP10.jpg]

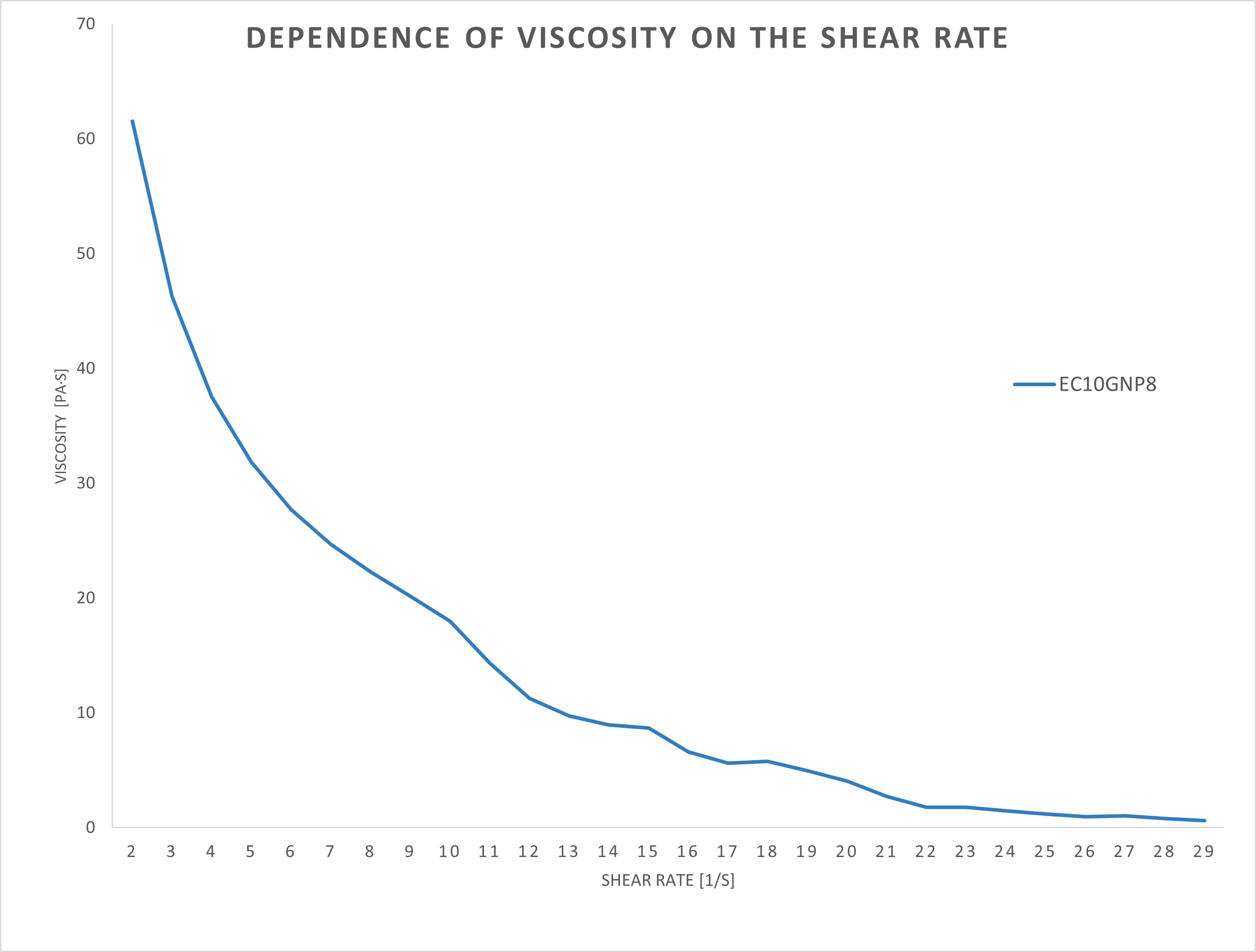

Supplement: Supplementary file 1 [file polymers-16-00686-s001.zip › rheology appendix/EC10GNP8.jpg]

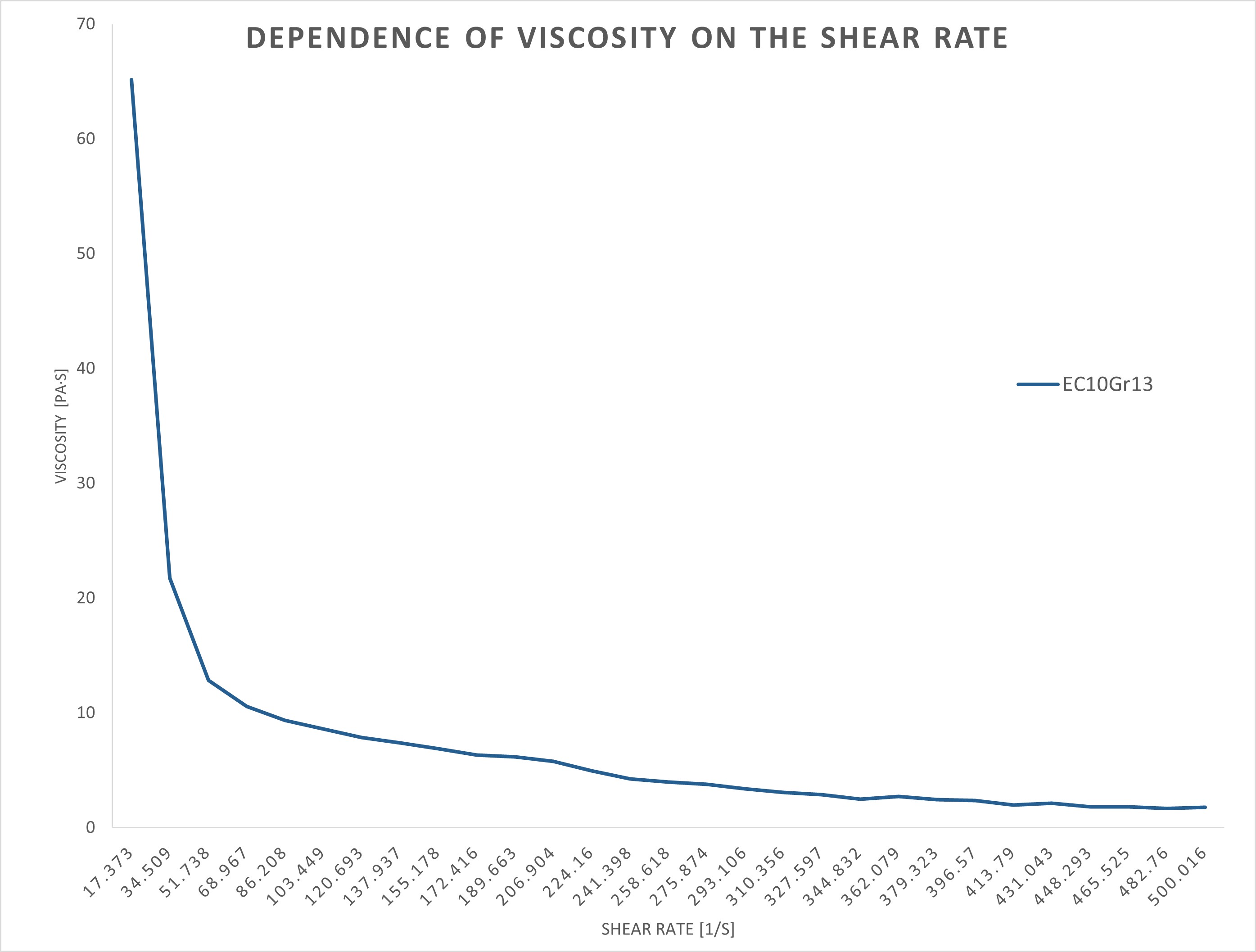

Supplement: Supplementary file 1 [file polymers-16-00686-s001.zip › rheology appendix/EC10Gr13.jpg]

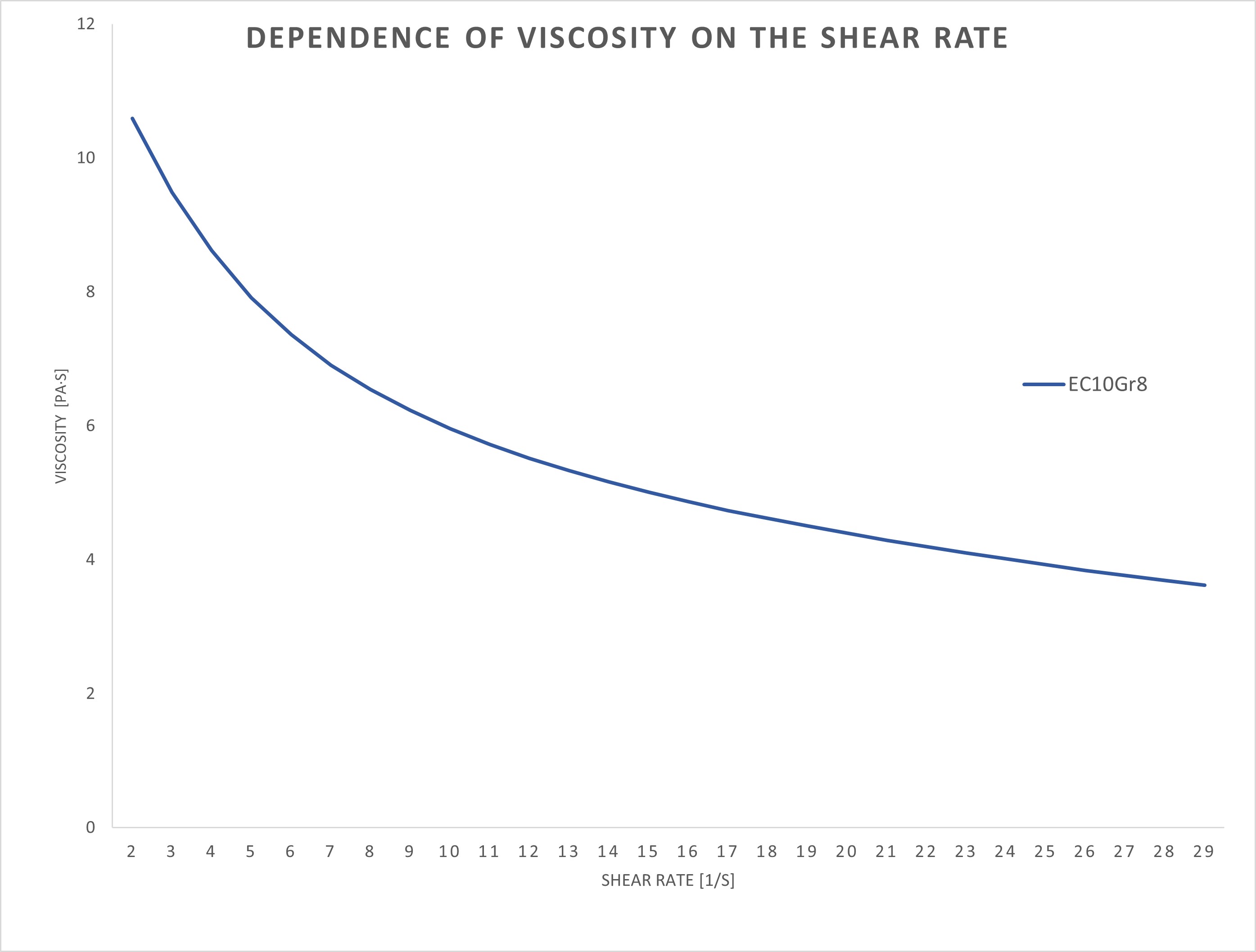

Supplement: Supplementary file 1 [file polymers-16-00686-s001.zip › rheology appendix/EC10Gr8.jpg]

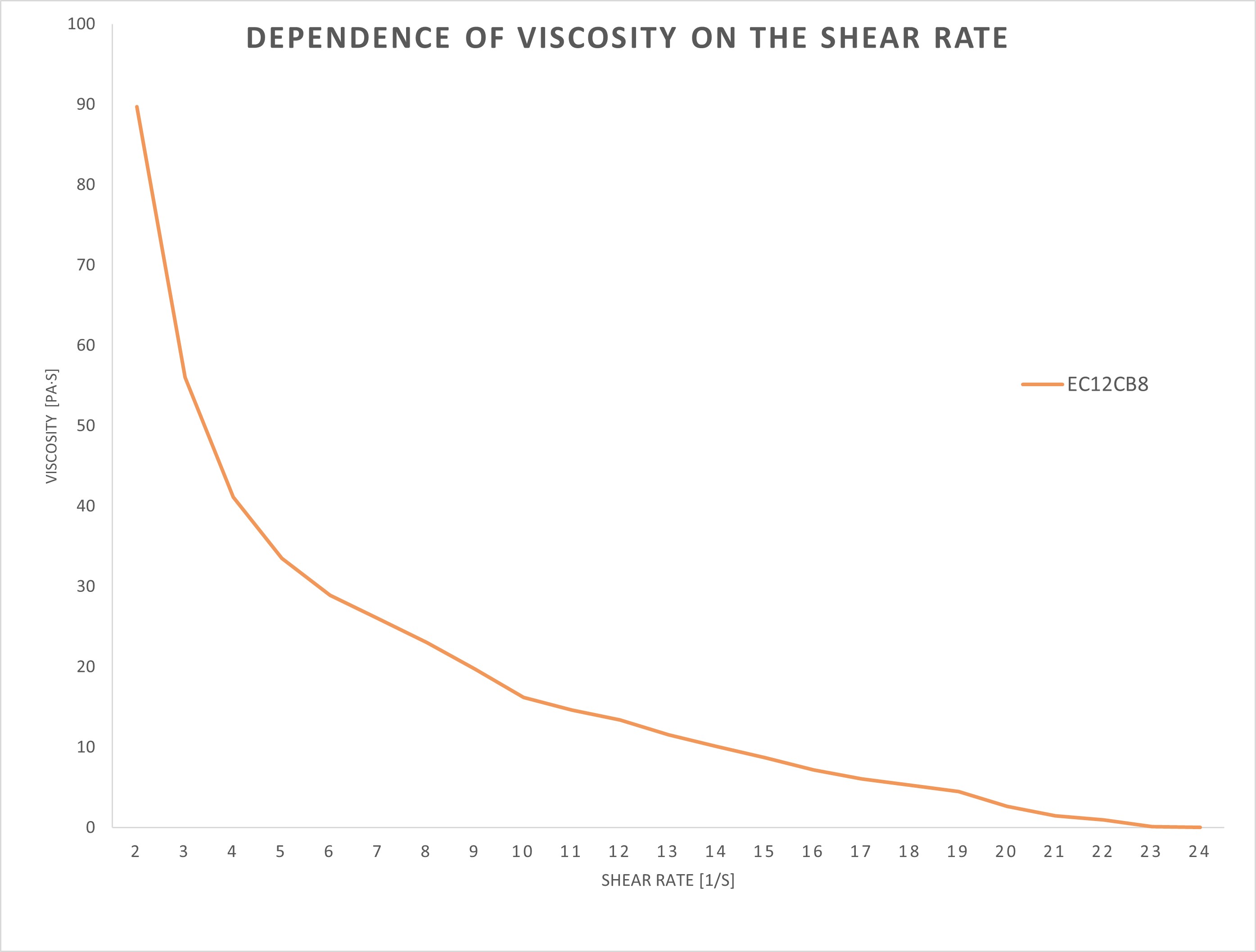

Supplement: Supplementary file 1 [file polymers-16-00686-s001.zip › rheology appendix/EC12CB8.jpg]

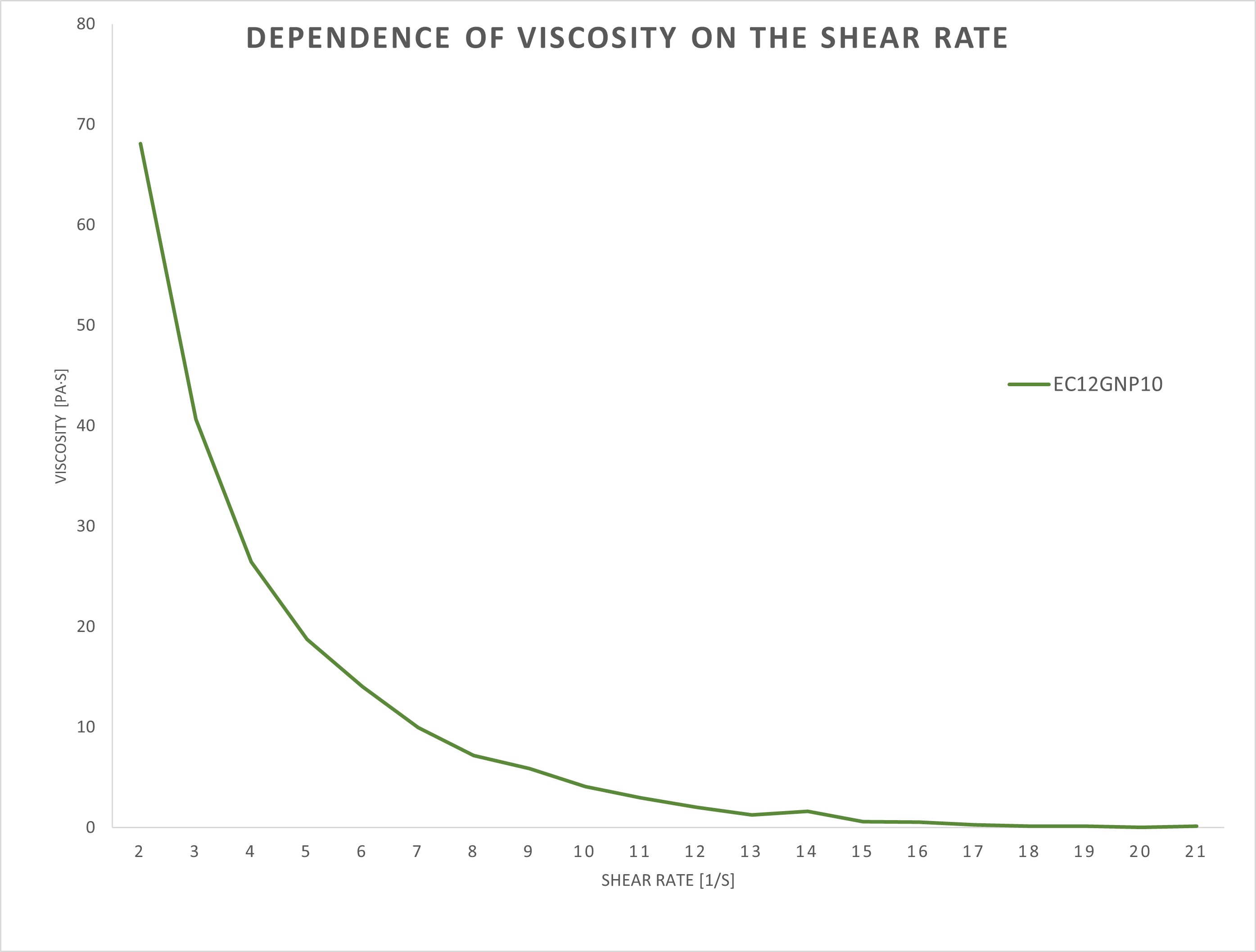

Supplement: Supplementary file 1 [file polymers-16-00686-s001.zip › rheology appendix/EC12GNP10.jpg]

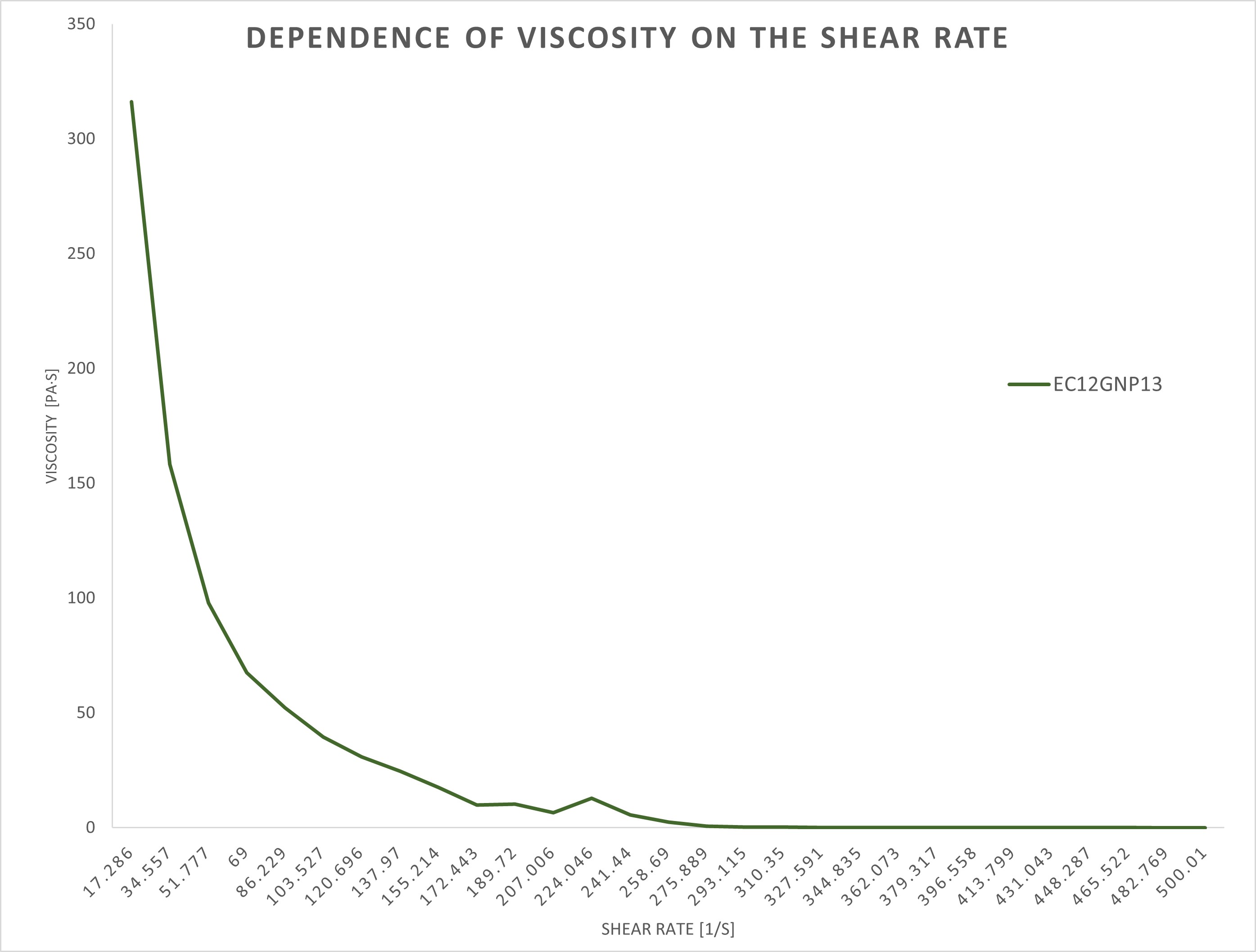

Supplement: Supplementary file 1 [file polymers-16-00686-s001.zip › rheology appendix/EC12GNP13.jpg]

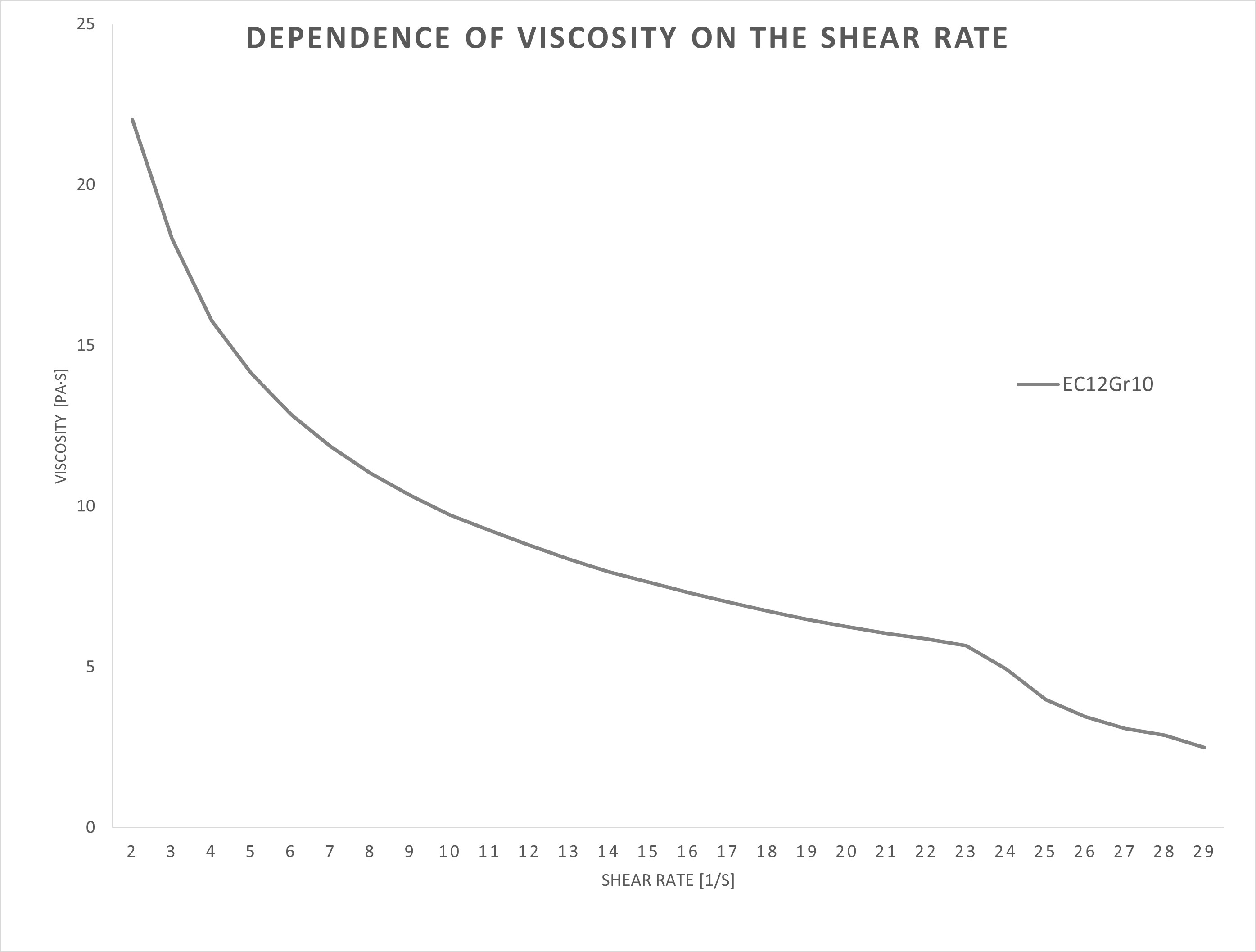

Supplement: Supplementary file 1 [file polymers-16-00686-s001.zip › rheology appendix/EC12Gr10.jpg]

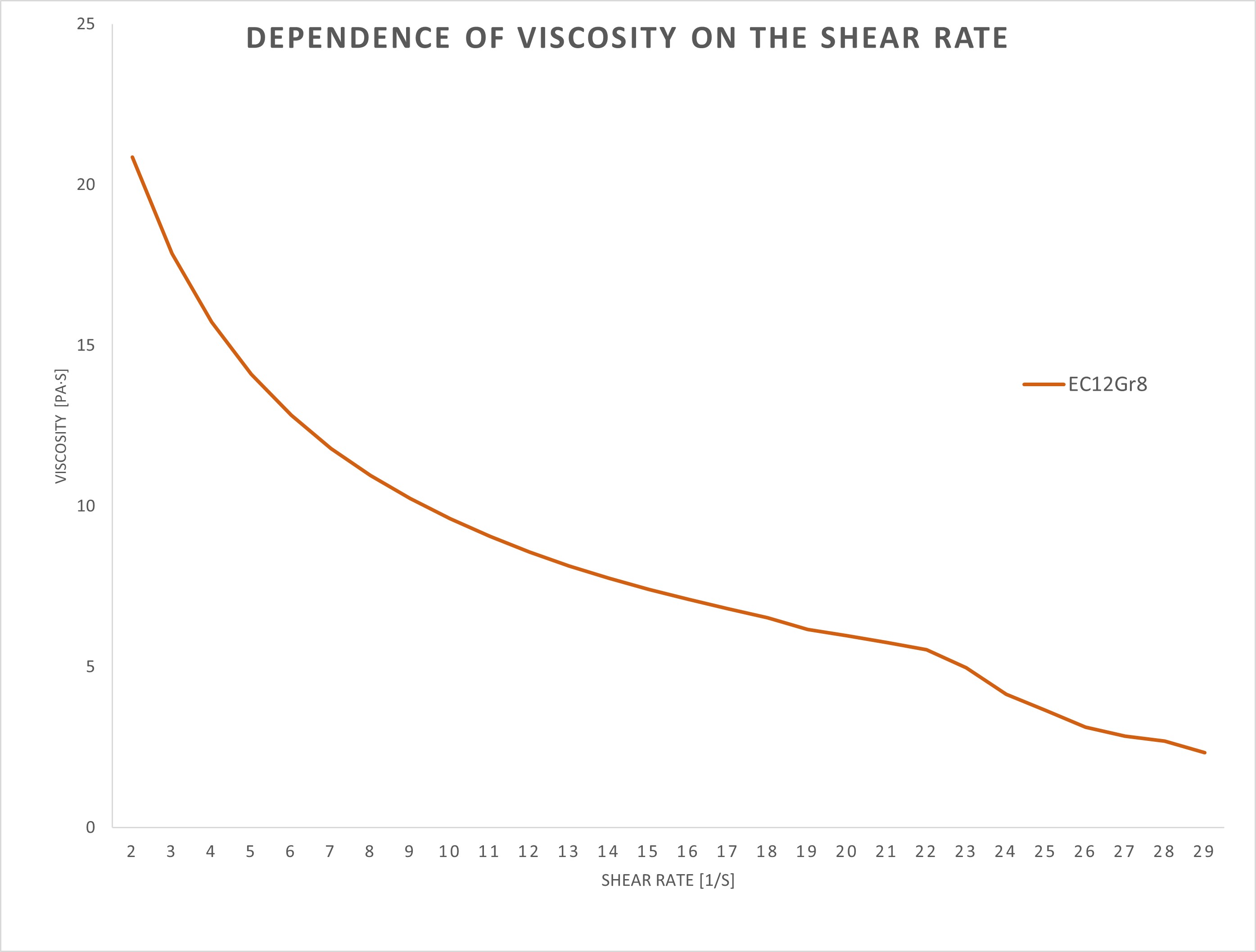

Supplement: Supplementary file 1 [file polymers-16-00686-s001.zip › rheology appendix/EC12Gr8.jpg]

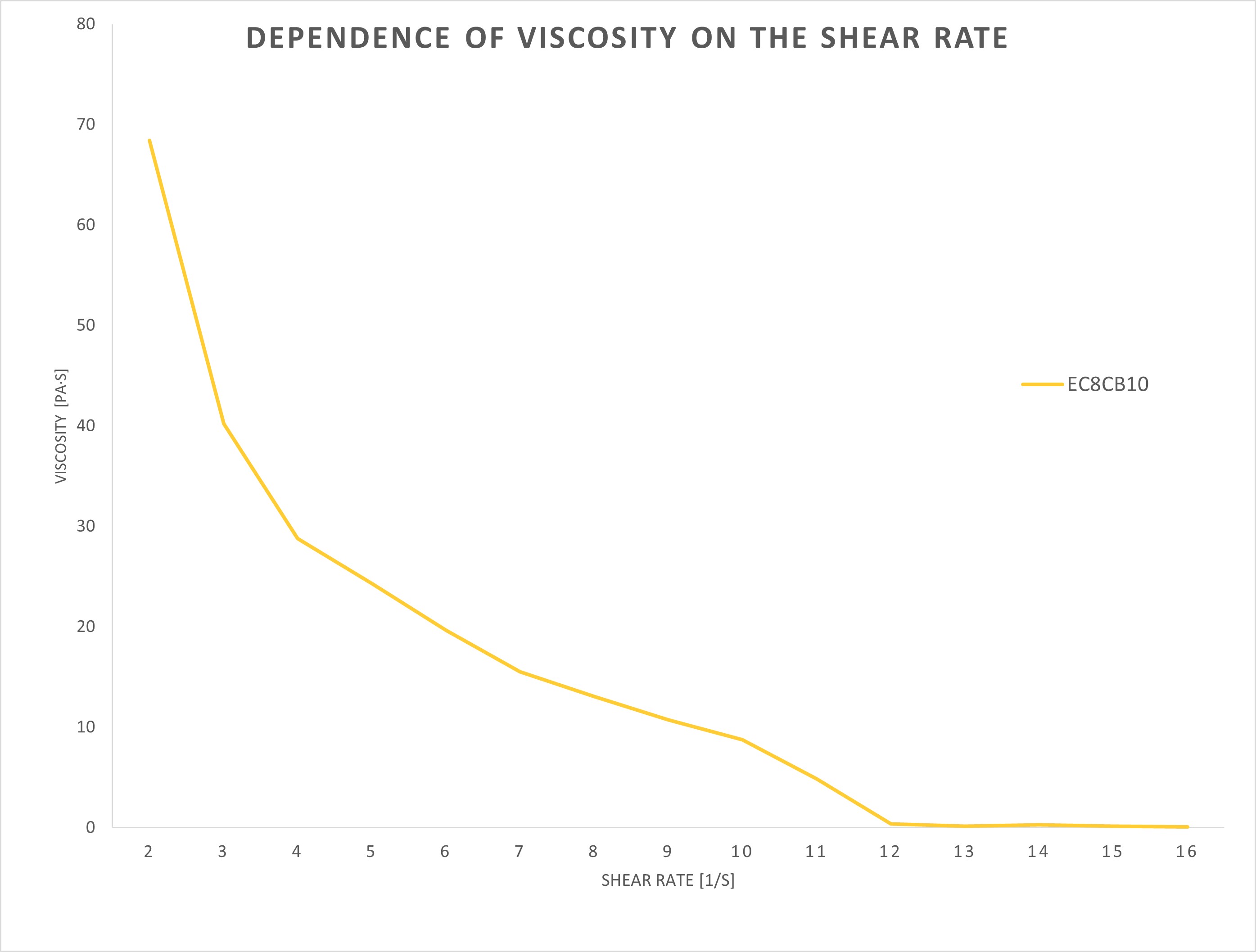

Supplement: Supplementary file 1 [file polymers-16-00686-s001.zip › rheology appendix/EC8CB10.jpg]

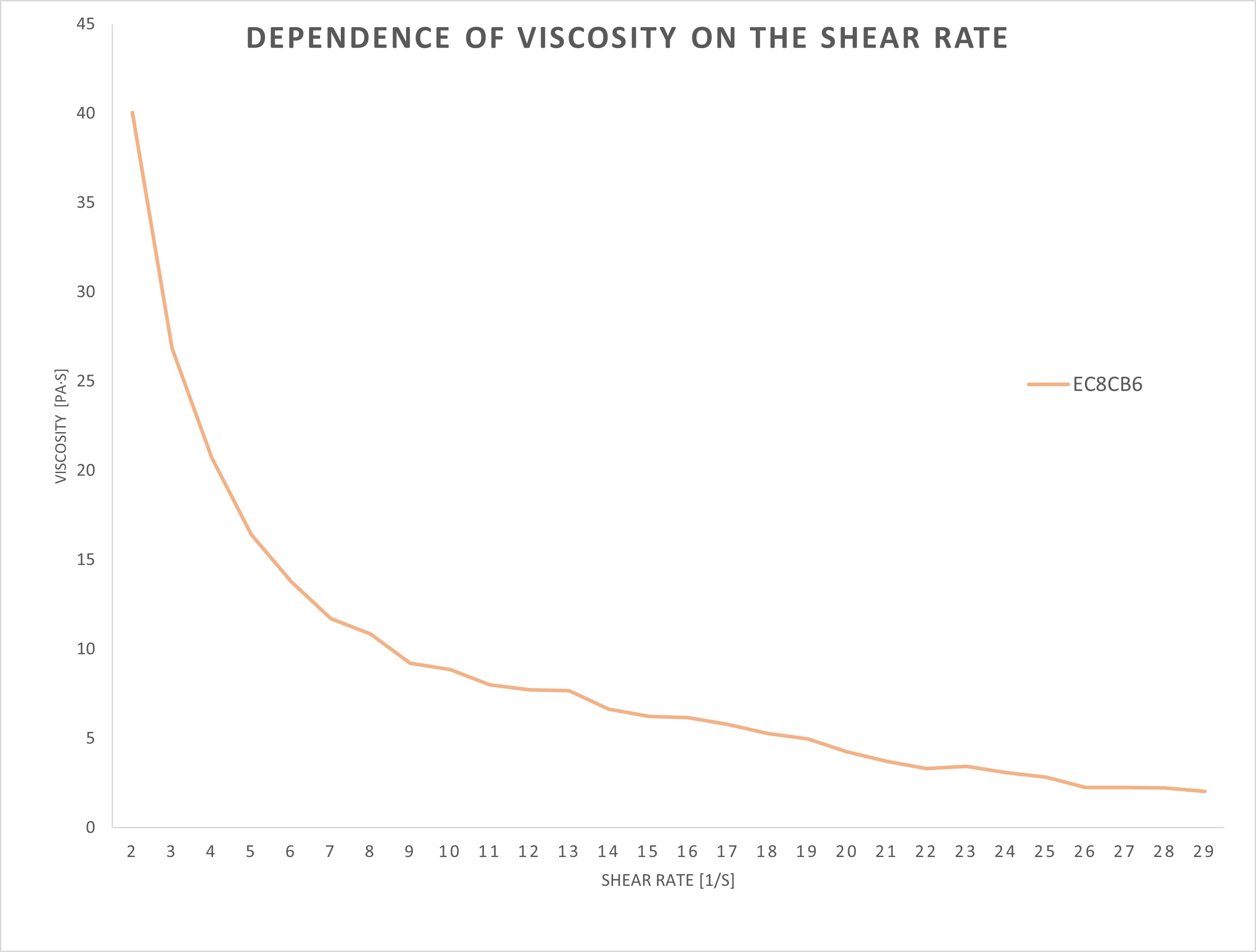

Supplement: Supplementary file 1 [file polymers-16-00686-s001.zip › rheology appendix/EC8CB6.jpg]

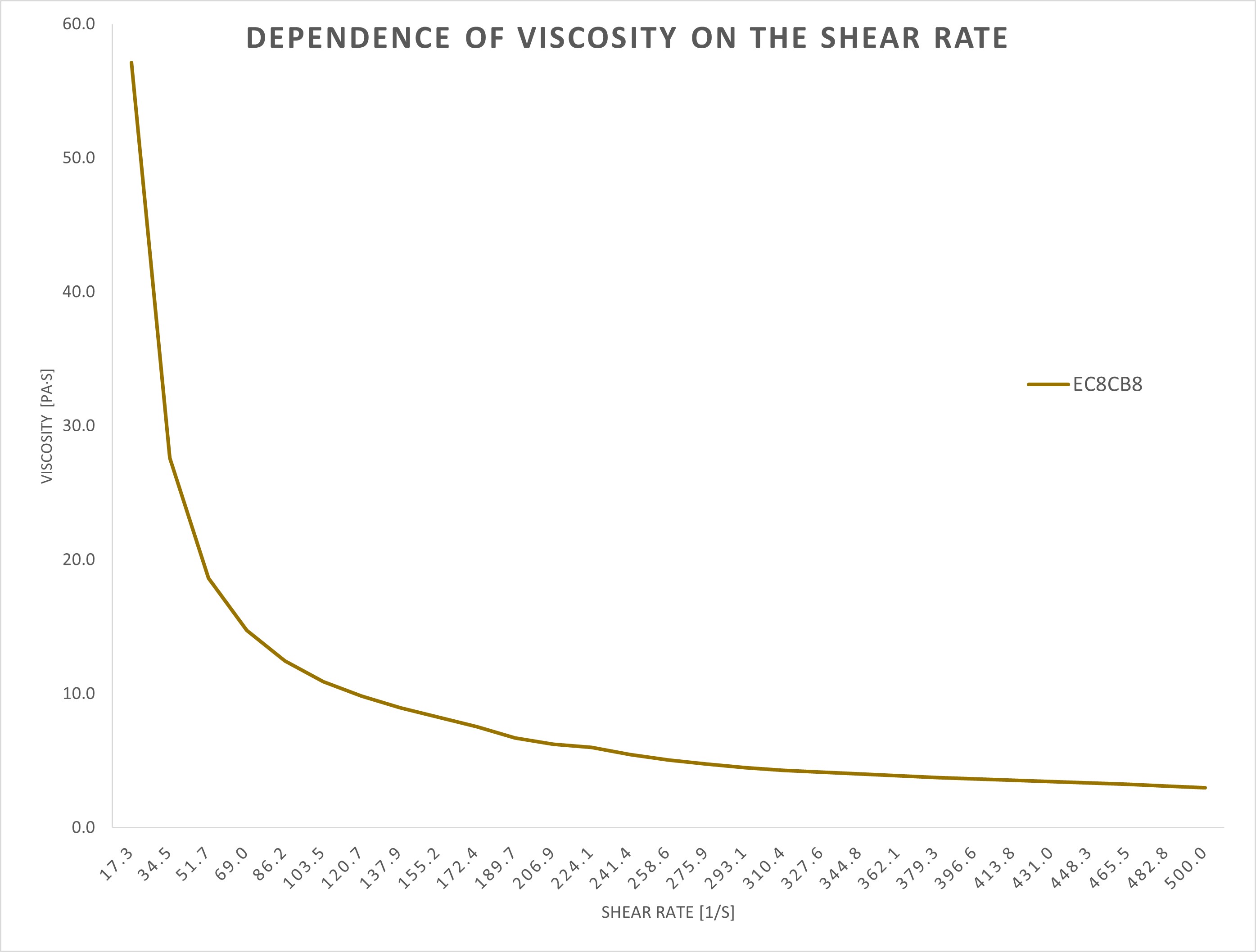

Supplement: Supplementary file 1 [file polymers-16-00686-s001.zip › rheology appendix/EC8CB8.jpg]

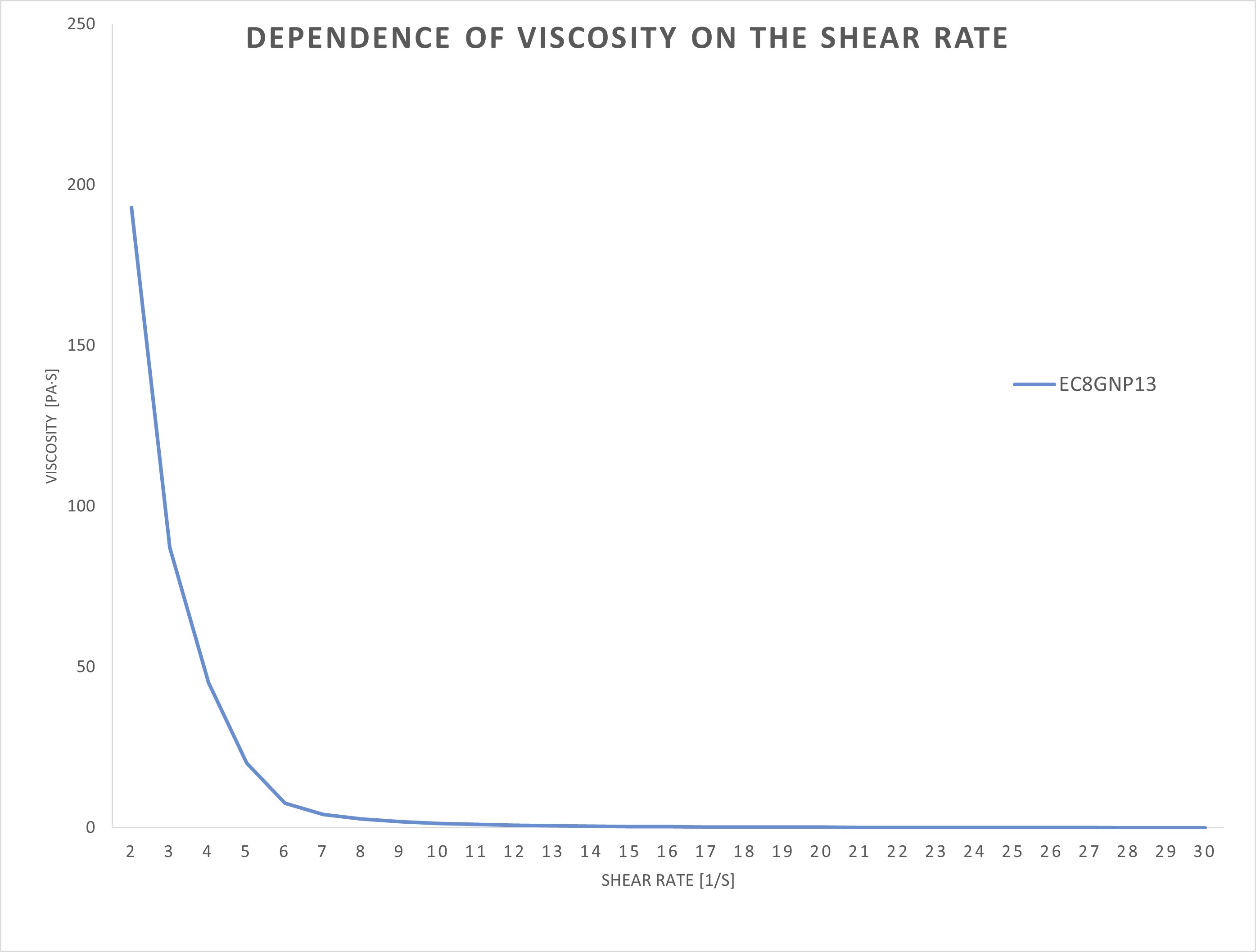

Supplement: Supplementary file 1 [file polymers-16-00686-s001.zip › rheology appendix/EC8GNP13.jpg]

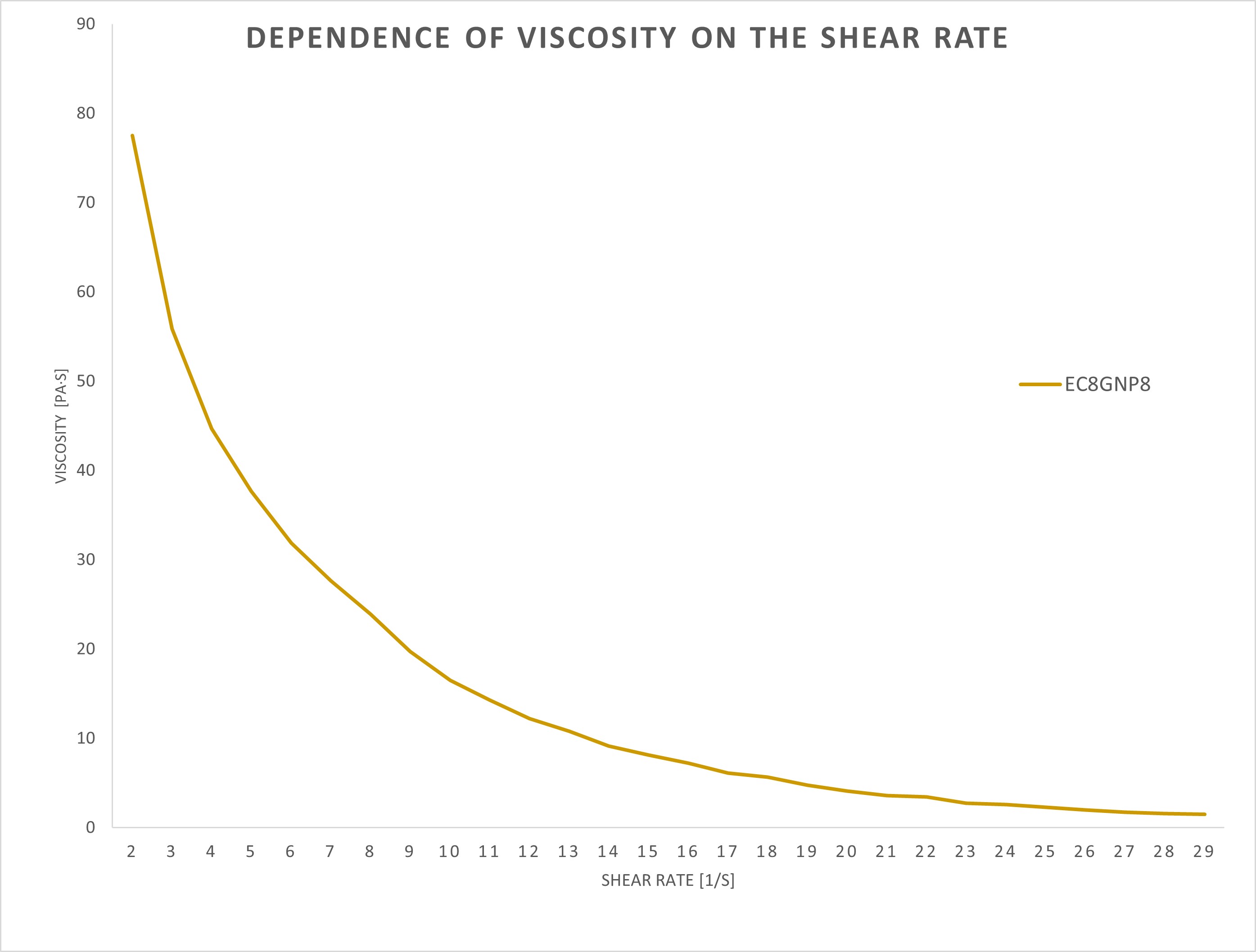

Supplement: Supplementary file 1 [file polymers-16-00686-s001.zip › rheology appendix/EC8GNP8.jpg]

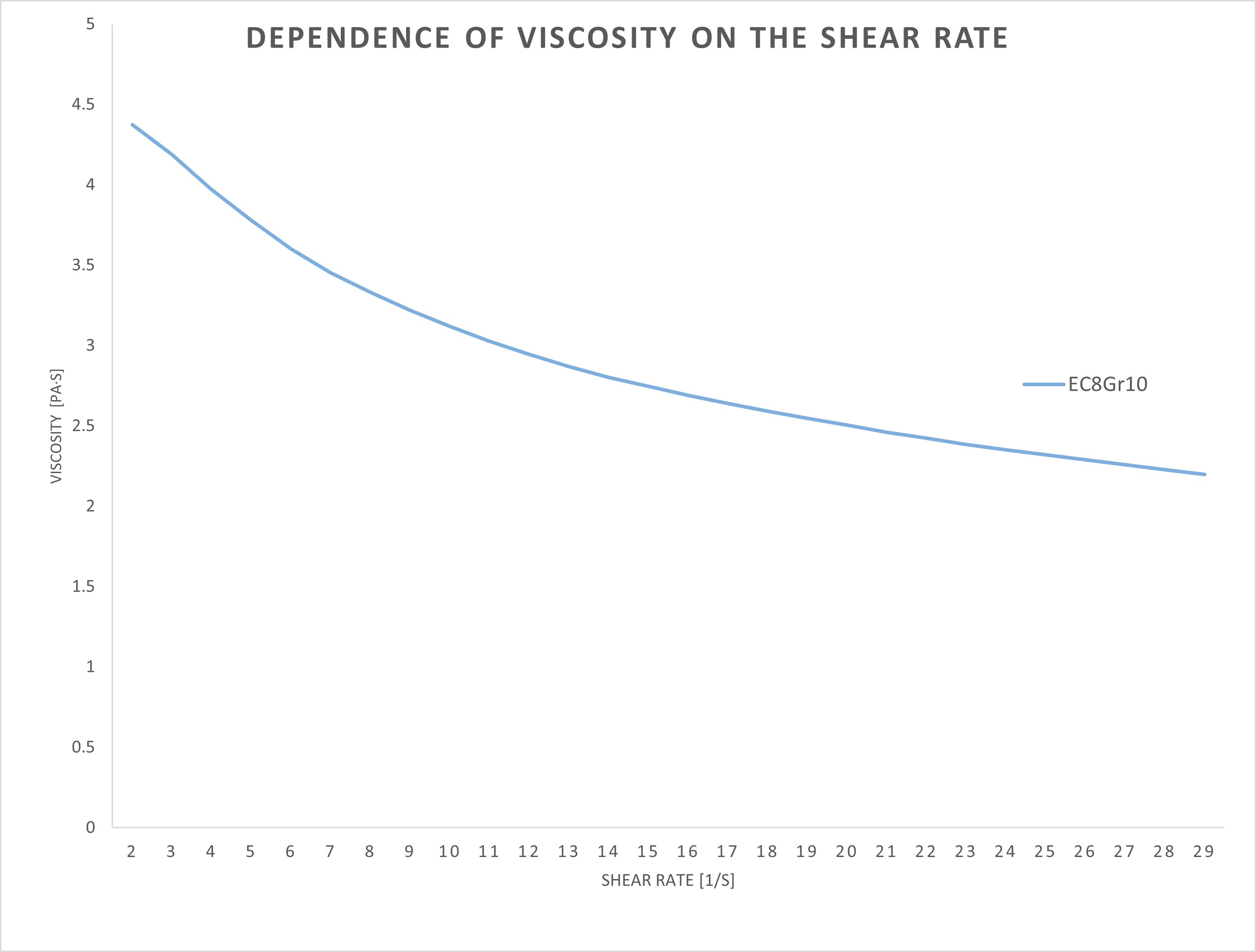

Supplement: Supplementary file 1 [file polymers-16-00686-s001.zip › rheology appendix/EC8Gr10.jpg]

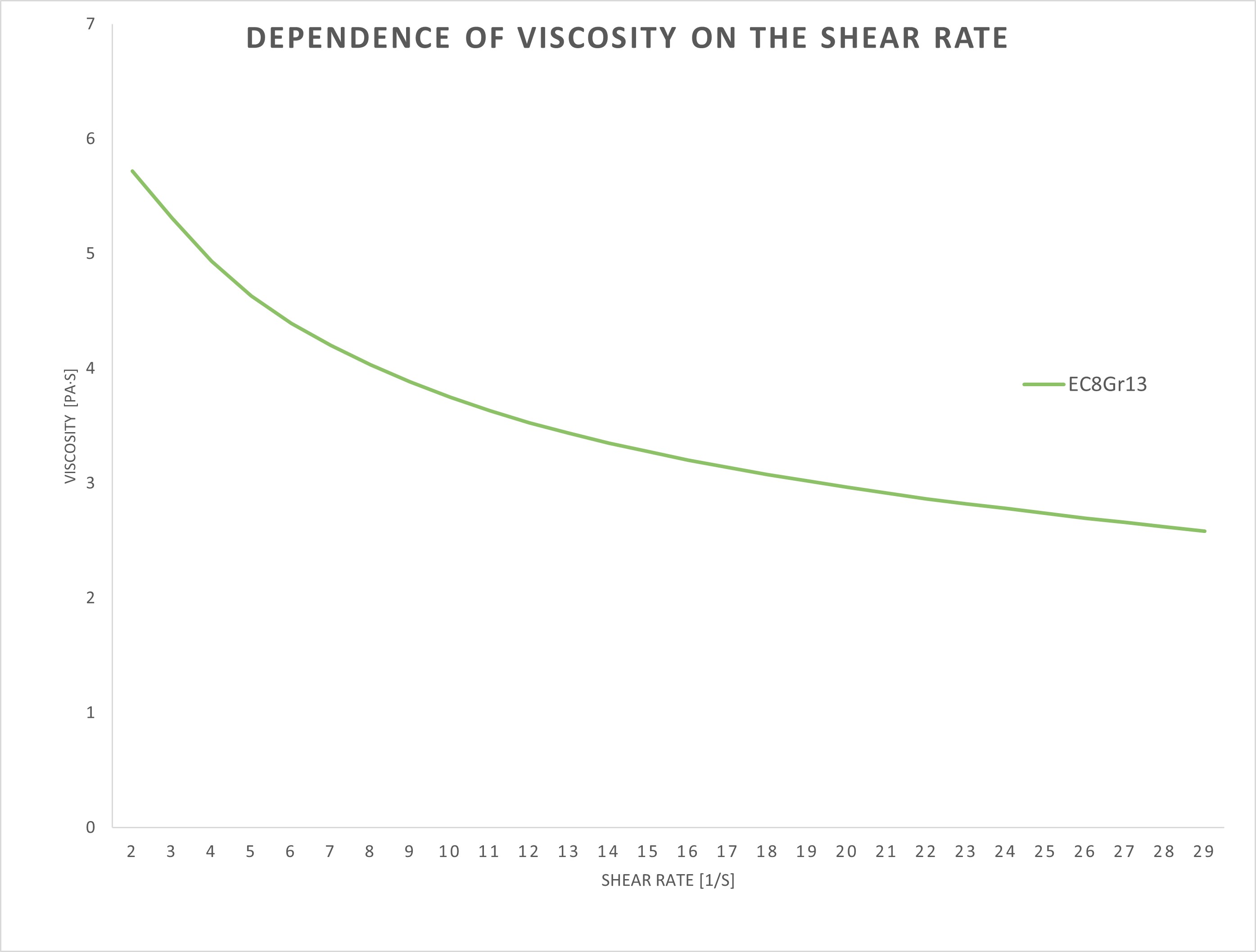

Supplement: Supplementary file 1 [file polymers-16-00686-s001.zip › rheology appendix/EC8Gr13.jpg]
